# Prevalence of hepatitis C in the adult population of Bulgaria: a pilot study

**DOI:** 10.1186/s13104-020-05158-3

**Published:** 2020-07-07

**Authors:** Ida Sperle, Stine Nielsen, Martyna Gassowski, Zlatina Naneva, Tanya Perchemlieva, Andrew Amato-Gauci, Matthias an der Heiden, Viviane Bremer, Elitsa Golkocheva-Markova, Kristiyan Hristov, Elena Kaneva, Yanita Simeonova, Tencho Tenev, Tonka Varleva, Erika Duffell, Ruth Zimmermann

**Affiliations:** 1 Department of Infectious Disease Epidemiology, Robert Koch Institute, Berlin, Germany; 2 Charité, Universitätsmedizin Berlin, Berlin, Germany; 3 Independent Consultant, Madrid, Spain; 4 Regional Health Inspectorate, Stara Zagora, Bulgaria; 5 European Centre for Disease Prevention and Control, Stockholm, Sweden; 6 National Reference Laboratory “Hepatitis Viruses”, Department of Virology, National Center of Infectious and Parasitic Diseases, Sofia, Bulgaria; 7 Ministry of Health, Sofia, Bulgaria

**Keywords:** Hepatitis C, HCV, Prevalence, General population, Prevalence survey, Bulgaria

## Abstract

**Objective:**

This study piloted a European technical protocol for conducting chronic hepatitis C prevalence surveys in the general population. The pilot study took place in the Bulgarian city of Stara Zagora in 2018, and results of setting up, conducting and evaluating the survey are presented.

**Results:**

A probability-based sample of the general adult population was drawn from the local population registry, stratified by age and sex. A sample size of 999 was calculated, and accounting for 50% non-response, 1998 registered invitation letters were sent. Venous blood samples and questionnaire data were collected by the Regional Health Inspectorate in Stara Zagora. Blood samples were tested for anti-HCV, and if reactive for RNA. 252 (21.6%) of the participants were included in the study. Mean age and sex distribution differed between the participants (55.9 years, 60.3% females) and the total sample (48.9 years, 53.4%). The weighted chronic HCV prevalence among participants was 0.9% [95% CI 0.2–4.2%]. The approach of only sending registered letters contributed to a low response rate, and more efforts are needed to reduce non-response, especially among men and younger age groups. Results of the evaluation were integrated in the final technical protocol.

## Introduction

The World Health Organization global strategy on viral hepatitis calls for elimination as a public health threat by 2030 [1] and national prevalence of chronic hepatitis C virus (HCV) infection is one of ten core indicators to be monitored [2].

HCV is primarily transmitted through infected blood and in European Union (EU) countries it mainly affects people who inject drugs (PWID) [3]. However, a higher prevalence may be found in birth cohorts of the general population (GP) exposed through nosocomial or transfusion-related transmission [4–7].

A recent systematic review found an anti-HCV prevalence in the GP in EU/European Economic Area (EEA) ranging from 0.1% (Belgium, Ireland and the Netherlands) to 5.9% (Italy) [3, 8]. Differences in prevalence between 16 countries with available estimates were difficult to interpret due to heterogeneous methodological approaches [3]. To address this, the European Center for Disease Prevention and Control contracted the Robert Koch Institute (RKI) from 2016 to 2019 to develop and pilot an evidence-based technical protocol with the aim to contribute to the standardisation of chronic HCV prevalence surveys in the GP. The protocol was developed in conjunction with an international and interdisciplinary expert panel and was published in March 2020 [9]. The stand-alone survey approach is one of three recommended approaches in the technical protocol and was piloted in the city of Stara Zagora, Bulgaria.

Stara Zagora is the sixth largest city in Bulgaria with an adult population of 120,849.^1^ The city has one of the country’s best economies [10]. Stara Zagora was selected as study site because of a strong collaboration between the Regional Health Inspectorate (RHI) and the Ministry of Health and a good laboratory testing infrastructure.

Robust data on HCV prevalence in Bulgaria are limited. One multi-centre study (1999–2000) among healthy volunteers in the five largest cities found an overall anti-HCV prevalence of 1.3% with a range from 1.1% in Stara Zagora and Plovdiv to 1.6% in Sofia [11]. Another study (2010–2011), found an 0.7% anti-HCV prevalence among outpatients in the Plovdiv Region [12].

This paper presents the results of the HCV prevalence pilot survey and reports on the feasibility of the protocol and key lessons learnt.

## Methods

A cross-sectional survey was undertaken to measure the chronic HCV prevalence (anti-HCV and RNA positive) in the adult GP (≥ 18 years) in Stara Zagora.

### Sampling

Based on an expected chronic HCV prevalence of 1% and a lower precision bound of 0.25%, a sample size of 999 was calculated. Accounting for an expected non-response rate of 50%, the total sample size was 1998.

A probability-based sample of the GP with current address in Stara Zagora, stratified by sex and six age groups (18–29, 30–39, 40–49, 50–59, 60–69, 70 + years), was drawn from the local population registry “Esgraon-TDS” [13, 14].

### Recruitment

Registered invitation letters (Additional file 1: S1a) were sent from the local population registry to the invitees. The first batch (400 letters) was sent two weeks prior to onset of data collection (05.09.2018). A reminder letter followed if no response within three weeks of sending the first letter (Additional file 2: S1b). If people were not home when the letter arrived, a note was delivered informing of the letter available to be collected at the local postal office.

The letters described the aims of the survey, selection of participants, opening hours and contact details of the study site and the availability of a mobile unit which could facilitate participation close to home and the incentive (coffee mug and pen) provided after participation. Voluntary participation, anonymity and confidentiality of data were underlined. A participant information leaflet (Additional file 3: S2) accompanied the letter providing more details about HCV, the survey, the importance of taking part, and that test results would be provided followed by linkage to care if HCV positive.

A local awareness campaign including posters in pharmacies and medical centers, announcements on RHI Facebook page and local press conferences to encourage participation was launched.

### Ethical approval, data protection

Persons in the sample were assigned an identification number and all identifiable information was kept at the local population registry. Participants provided written informed consent. Original data were kept at the RHI and copies were sent to RKI via an online server allowing an encrypted secure transfer. The survey protocol was approved by the local ethics committee established at the RHI.

### Data collection

Data were collected from 5 September to 16 November 2018, Monday–Friday: 8:30 am–7:00 pm and Saturday: 8:30 am–1:30 pm at the RHI, and on Saturdays also in a mobile unit.

On site, participants self-completed a questionnaire on socio-demographics, HCV testing history, knowledge of HCV status and risk factors. Basic sociodemographic information on non-responders who called to decline participation was collected over the phone.

Venous blood samples were tested for HCV antibodies [Bioelisa, antibody HCV 4th generation (by Biokit)] at the RHI laboratory. Anti-HCV reactive samples were tested for RNA (Additional file 4. S3) (HCV Real Time PCR, Abbott, USA) at the National Reference Laboratory “Hepatitis viruses”, Sofia. RNA negative samples were tested by immunoblot (Inno-Lia HCV score, Fujirebio, Belgium) to confirm the positive anti-HCV result.

During a face-to-face consultation at the RHI a medical doctor informed participants about their test results. Those with chronic HCV were referred to a gastroenterologist in the hospital of Stara Zagora where liver function was assessed and treatment initiated in line with national guidelines.

### Data analysis

Double data entry was performed using EpiData (version 4.4.2.1), and analyses in STATA 15. Descriptive analysis was performed for all variables. T-test was used to compare the mean age among the participants and the total sample, and chi-squared test was used for sex with the statistical significance defined as *p *value < 0.05. Chronic HCV prevalence was calculated as crude and weighted estimates with 95% confidence intervals (CI). For the latter, we applied post-stratification weights according to age and sex to adjust for non-response.

### Evaluation of the draft technical protocol

Indicators were developed and transformed into a questionnaire with 10 main questions covering objectives of the survey, methodology, time, structure, coordination and collaboration, ethical approval, data protection, staff and budget to be completed by the RHI study team. During a 2 day evaluation workshop recommendations for improvement of the protocol were discussed with 14 survey staff members.

## Results

**Fig. 1:**
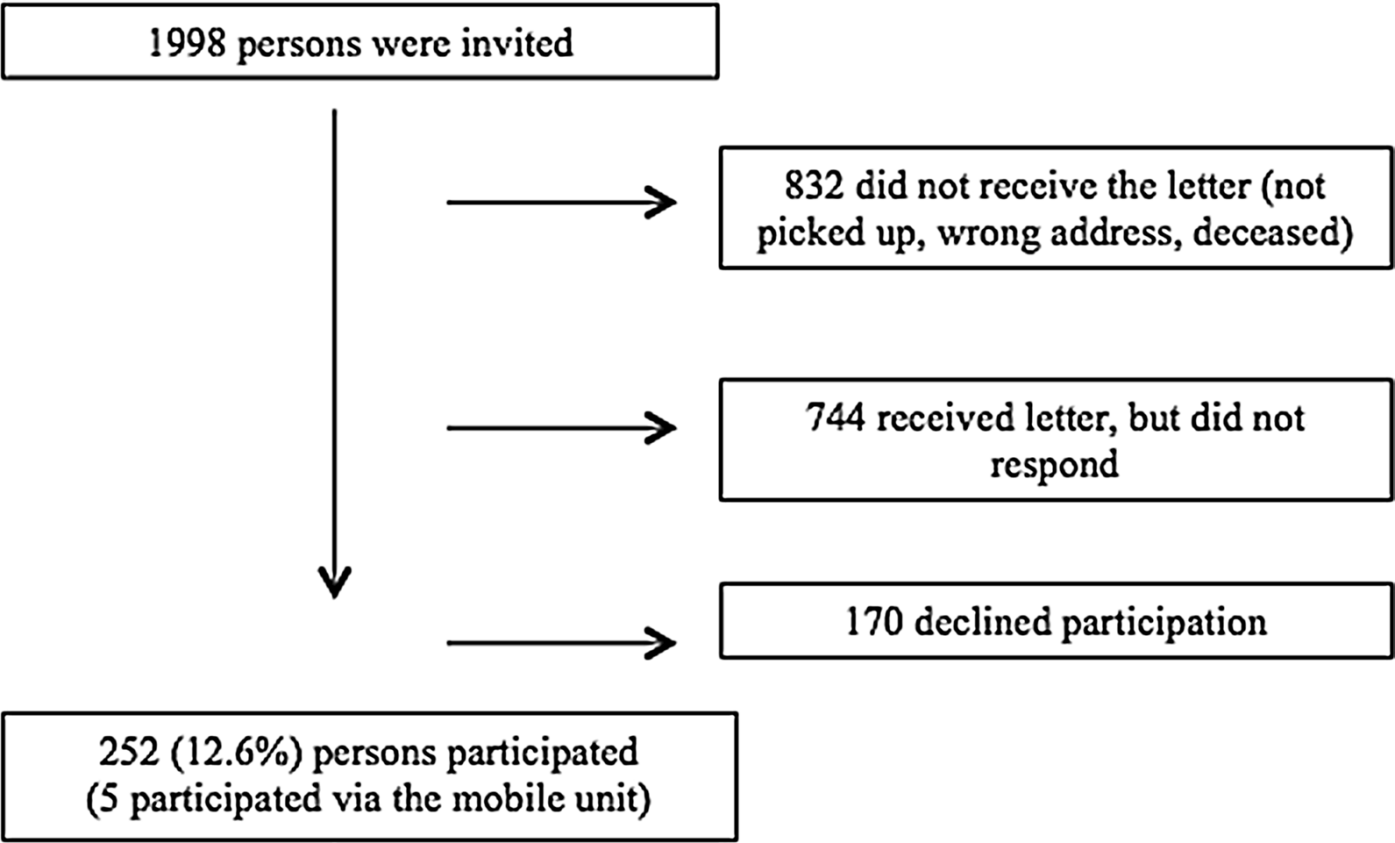
Flowchart of participation

Of 1998 invited people, 1166 received the invitation letter of which 252 participated (21.6%) (Fig. 1).

Of the 832 who did not receive the letter, two were deceased. The rest did either not pick up the letter or were registered with a wrong address.

### Non-participation analysis

Among 170 declining participation, 155 (91.2%) provided reasons for non-participation (Table 1).

**Table 1:** Reasons for non-participation

Reason for non-participation (n = 155)	n (%)
Generally dislike surveys	41 (26.5%)
Living abroad	37 (23.9%)
Not interested	27 (17.4%)
No time	26 (16.8%)
Too ill	22 (14.2%)
Live too far away	6 (5.8%)
Known HCV negative	5 (3.2%)
No suitable appointment	2 (1.3%)
Blood donor	2 (1.3%)
Fear of needles	2 (1.3%)
Got tested in 2018	1 (0.6%)
Known HCV positive	0 (0%)
Do not wish to provide a reason	2 (1.3%)

The age and sex distribution among participants differed significantly from the total sample (n = 1998). The mean age for participants was 55.9 years versus 48.9 years for the total sample (*p* < 0.0001). There were 60.3% females among participants versus 53.4% in the total sample (*p* < 0.0001) (Additional file 4: S3).

### Survey participants characteristics

Participants’ mean age was 55.9 years (18–95 years) (Table 2) (Additional file 4: S3).

**Table 2:** Sociodemographic characteristics of participants (n = 252)

Sociodemographic characteristics	n (%)
Sex	
Female	152 (60.3%)
Male	100 (39.7%)
Ethnicity	
Bulgarian	248 (98.8%)
Roma	2 (0.8%)
Other	1 (0.4%)
Missing	1 (0.4%)
Highest level of education	
Elementary education	1 (0.4%)
Primaryeducation	11 (4.4%)
Secondary education	122 (48.4%)
Higher education	118 (46.8%)

### Prevalence of HCV

Two participants were both anti-HCV and HCV-RNA positive, crude chronic HCV prevalence: 0.8% [95% CI 0.2–3.1%], weighted prevalence: 0.9% [95% CI 0.2–4.2%].

### Factors associated with HCV

Among the 252 participants, the most frequently reported factors associated with HCV were surgery under general anesthesia (64.1%), followed by blood transfusion before 1992 (11.7%) (Additional file 4: S3). One of the two HCV positive participants reported having injected drugs, the other did not report any known factors associated with HCV.

### Results from the evaluation

Sufficient staff training was provided and the protocol was evaluated as useful and understandable. The extended opening hours helped accommodate participation of people who work, whereas the mobile unit was less utilised. Planning took more time than expected (one full-time equivalent staff for nine months) particularly on administrative tasks and data protection issues. In total, 19 RHI staff were involved in the data collection.

## Discussion

We performed a cross-sectional survey with the aim to pilot the draft technical protocol, assess its feasibility and to generate an HCV prevalence estimate in the GP of Stara Zagora. As the Data Protection Commission denied RHI access to contact information for the invited participants, the initially planned recruitment strategy (involving house-visits to non-responders) was changed, allowing only recruitment via letters which resulted in not reaching the calculated sample size (n = 999). As consequence a low precision for the HCV prevalence estimate, for which reason weighting was performed to adjust for non-response. The prevalence may under- or overestimate the true prevalence due to the failure of including persons less likely to participate. Low participation and selective non-participation cause bias to survey results [15, 16]. Lower socio-economic status, a poorer health profile and higher mortality have previously been found among non-participants compared to participants [17, 18]. In this survey, non-participation was more frequent among men and younger age groups. Higher participation among women and older age groups corresponds with findings from other similar surveys [19]. Reasons for non-response are likely multifaceted, and may differ depending on sex and age group.

Of the 170 people who actively declined participation, 41 (24%) lived outside Stara Zagora. It is plausible that a similar proportion among the 832 who did not receive the letter also migrated to other cities or countries e.g. for work. This indicates that the sampling frame was not up-to-date which is a key requirement for surveys [20].

More efforts are needed to reduce non-participation, but their effectiveness may differ between settings [21, 22]. In a German Health Survey phone calls and house visits increased participation from 37 to 49%, with greater effect among younger persons, males and non-Germans [23]. In Finland, SMS reminders have shown a positive effect [24]. We used registered letters allowing monitoring of whether letters were received or not, but in Bulgaria registered letters are often associated with “bad news” (e.g. fines or unpaid taxes). In addition to the inconvenience of collecting the letter at the postal office, this may explain why many letters were not picked up. Also, recruitment via mobile unit might have worked better if addresses had been available to RHI staff enabling them to then proactively visit people.

The incentives provided were well accepted, but different incentives for different age groups might have impacted positively on the response rate. In Germany gift vouchers work well.^2^ Some studies have shown that monetary incentives are preferred [25] whereas in others, participants considered them to impose an unwanted commercial feature and undermine confidence in the survey [26]. Pre-survey qualitative assessments, e.g. focus groups, are recommended to identify the most effective measures to increase participation [27].

The HCV prevalence weighted for age and sex was 0.9% [95% CI 0.2–4.2%], and similar to that found in the 1999–2000 study among healthy volunteers in Stara Zagora [1.05% (anti-HCV)] [11]. Although non-response bias cannot be ruled out, the use of weights likely reduced non-response bias.

Two thirds of participants reported having been exposed to risk factors for HCV infection, with surgery under general anesthesia being reported by two thirds of participants. Nosocomial transmission was the second most common transmission-route among acute HCV cases in 2017 in the EU/EEA (17%) [28]. In Bulgaria, recipients of a transfusion of unscreened blood (prior to 1992) are a key risk group. Recent reports of breaches of infection control procedures also indicate that iatrogenic transmission may be a current risk factor for HCV in Bulgaria [29, 30], however in our sample, even in those reporting potential exposure, none tested positive for HCV. Two participants reported injecting drug use, and one of them tested HCV positive. The highest rates of chronic HCV prevalence in Europe are found among PWID ranging from 13.8 to 84.3% (anti-HCV) [31]. Studies in Bulgaria have found high levels of HCV transmission among PWID and other groups [32], with one study in Sofia reporting 73.9% of 773 PWID being anti-HCV positive [33]. GP surveys are not ideal to collect representative data on PWID. Other recruitment strategies are needed for this vulnerable population [34].

Self-reported data may be prone to social-desirability bias. Questions about drug use, imprisonment and previous test results are sensitive and people may tend to provide answers perceived as more socially acceptable. Social-desirability bias however is often reduced when the questionnaire is self-administered [35]. Recall bias might also have played a role in this survey.

Our survey methodology was found to be feasible, understandable and helpful in providing a step-by-step approach on how to implement a HCV prevalence survey in the GP. Despite the low response rate, the survey approach was found to be useful in estimating the prevalence but also resource intensive in terms of time, staff and costs. All lessons learnt were included in the final version of the technical protocol [9].

The technical protocol targets the GP [9], and estimating the prevalence among the GP is one step needed to estimate the overall national HCV burden. The technical protocol provides an opportunity to improve the availability of reliable and robust data to describe the HCV epidemiology and contribute to monitoring progress towards the elimination of viral hepatitis.

## Limitations

The main limitation in this study is the low response rate which reduced the reliability and validity of the results. Therefore, we cannot draw conclusions regarding HCV prevalence in the GP in Stara Zagora.

## Supplementary information

**Additional file 1. S1a:** First invitation letter

**Additional file 2. S1b:** Second invitation letter

**Additional file 3. S2:** Participant information leaflet

**Additional file 4. S3:** Additional tables and figures

## Data Availability

The study protocol and the datasets analysed are available from the corresponding author upon request.

## Abbreviations

Anti-HCV: Antibody to hepatitis C virus
CI: Confidence intervals
EU/EEA: European Union/European Economic Area
GP: General population
HCV: Hepatitis C virus
HCV-RNA: Hepatitis C virus ribonucleic acid
PWID: People who inject drugs
RHI: Regional Health Inspectorate
RKI: Robert Koch Institute

## Footnotes

1 Registered as currently living in Stara Zagora as of 15 March 2018 (Information from the Local Population Registry, “Esgraon-TDS”, in Stara Zagora)

2 Dr. Antje Gößwald, RKI, personal communication, April 2, 2019
