# Supplementary material for: Prevalence of hepatitis C in the adult population of Bulgaria: a pilot study
Source: BMC Res Notes. 2020 Jul 7;13:326. doi: 10.1186/s13104-020-05158-3 (PMC7341663; doi:10.1186/s13104-020-05158-3)
Supplement: Supplementary file 2 — Additional file 2. S1b: Second invitation letter [file 13104_2020_5158_MOESM2_ESM.pdf]

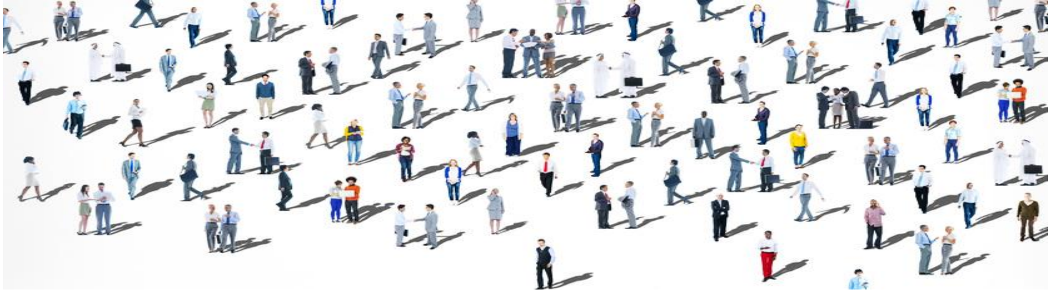

ROBERT KOCH INSTITUT

Street and number: *[Participant ID]*
Entrance:
Floor:
Apartment:
Stara Zagora

Reminder of invitation to participate in the SPHERE-C survey

Dear Mr. /Ms. *[first name, last name]*

This is a gentle reminder and follow-up to the previous letter inviting you to participate in a survey, which will study the prevalence of hepatitis C in Stara Zagora. As participant you will be offered free hepatitis C testing and will get to know your status, so that if positive, you can get access to the needed medical care. Further, we will offer a small gift as a token of our appreciation for your participation.
With your help, we will be able to provide an estimate of the hepatitis C prevalence in Stara Zagora, Bulgaria, which will be used for further public health planning.

You were randomly selected from the local population register in Stara Zagora to represent the adult population (18 years and older) in Stara Zagora.

When taking part in the survey, you will be asked to complete a short questionnaire after which a blood sample will be collected. It takes around 30-40 minutes in total to take part in the survey.

The success of the survey requires a high participation rate. Your participation is therefore very important, also if you are already aware of your hepatitis C status. Your participation cannot be replaced by someone else. Your participation is voluntary. All information collected during the survey, will be handled confidentially. You can find answers to questions regarding the survey in the enclosed information leaflet.

Please call **042 604151** or **042 602468** to make an appointment or visit the
Regional Health Inspectorate (ul. "Stefan Karadzha" 10, 6001 кв. Опълченски, Stara Zagora) within the next two weeks, if you would like to participate.

Working hours:	Monday-Friday:	8.30 - 19.00
	Saturdays:	8.30 - 13.30

The survey runs from 5 September – 16 November 2018.

If you are unable to come to the Regional Health Inspectorate, there is an option of getting tested in a mobile unit which can come to your address. Please call 042 604151 or 042 602468 to learn more about this opportunity.

If you cannot participate, we will appreciate very much if you will call one of the numbers above and let us know and then you will not be contacted again.

Remember to bring this invitation letter and a photo identification card for the appointment.

The Survey team thanks you for your collaboration.

Sincerely,

____

Zhivko Todorov

Mayor of Stara Zagora
