# Supplementary material for: Prevalence of hepatitis C in the adult population of Bulgaria: a pilot study
Source: BMC Res Notes. 2020 Jul 7;13:326. doi: 10.1186/s13104-020-05158-3 (PMC7341663; doi:10.1186/s13104-020-05158-3)
Supplement: Supplementary file 3 — Additional file 3. S2: Participant information leaflet [file 13104_2020_5158_MOESM3_ESM.pdf]

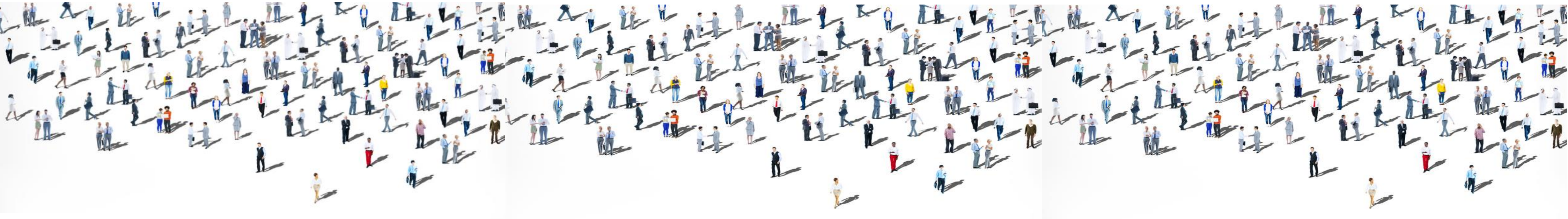

What will happen to the results of the survey?

Your personal information and results of the hepatitis C test are safely stored and only accessed by the study coordinator.

For analysis, the data will be anonymised, which means that each name will be assigned a number.

The results of the survey will be used to write a report on the hepatitis C prevalence in Stara Zagora.

For further Information and if questions please do not hesitate to reach out to:

The Regional Health Inspectorate

ul. "Stefan Karadzha" 10
6001 кв. Опълченски, Stara Zagora

Phone: 042 604151 & 042 602468
E-mail: rzistz@rzistz.org

SPHERE-C

Participant Information Leaflet

ecdc EUROPEAN CENTRE FOR DISEASE PREVENTION AND CONTROL

ROBERT KOCH INSTITUT

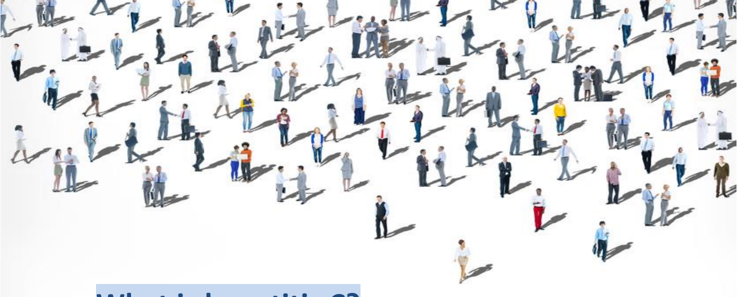

What is hepatitis C?

Hepatitis C is a contagious liver disease that ranges in severity from a mild illness lasting a few weeks to a serious, lifelong illness that attacks and damages the liver permanently.

It results from infection with the Hepatitis C virus, which is spread primarily through contact with the blood of an infected person. People who are tested positive to hepatitis C can be treated and cured. In most European countries less than 1% of the adult population is infected (a person may be infected without having any clinical manifestations of the disease). The blood test is the only method that detects the disease in this case.

What is SPHERE-C?

The SPHERE-C project was launched by the European Centre for Disease Prevention and Control (ECDC),

and is carried out by the Robert Koch-Institute, the national public health institute in Germany,

in collaboration with partners at the Municipality and the Regional Health Inspectorate in Stara Zagora and the Ministry of Health, Bulgaria.

The survey in Stara Zagora runs from 05.09.-16.11.2018.

What is this survey for?

The survey will give us information about the number of people who are living with hepatitis C in Stara Zagora, Bulgaria.

This will have large public health implications, as a current estimate enables better planning the overall response to hepatitis C.

Why was I invited?

Your name and address was randomly selected together with about 2000 other people living in Stara Zagora.

Do I have to take part?

Participation is entirely voluntary and if you decide not to take part, let us know via telephone and then you will not be contacted again.

What will happen if I decide to take part?

If you decide to take part, please call the number above to make an appointment. You will then be asked to sign an informed consent form and to fill in a short questionnaire. Thereafter a nurse will draw a blood sample which will be tested for hepatitis C.

You will receive a letter with your ID number and a specific date for receiving the test result. You will be asked to come to the Regional Health Inspection to get it.

If you are positive, you will be counselled in terms of next steps and medical care needs.

You will receive a small gift after participating in the survey as a token of appreciation.

Why is it important that you take part?

You will be offered free hepatitis C testing and will get to know your status, so that if positive, you can get access to the needed care. Further, with your help, we will be able to provide an estimate of the hepatitis C prevalence in Stara Zagora, Bulgaria, which will be used for further public health planning.

For this purpose, it is important that you take part even if you know your hepatitis C status.
