# Supplementary material for: Prevalence of hepatitis C in the adult population of Bulgaria: a pilot study
Source: BMC Res Notes. 2020 Jul 7;13:326. doi: 10.1186/s13104-020-05158-3 (PMC7341663; doi:10.1186/s13104-020-05158-3)
Supplement: Supplementary file 4 — Additional file 4. S3: Additional tables and figures [file 13104_2020_5158_MOESM4_ESM.docx]

# Supplementary Materials

**Testing algorithm**


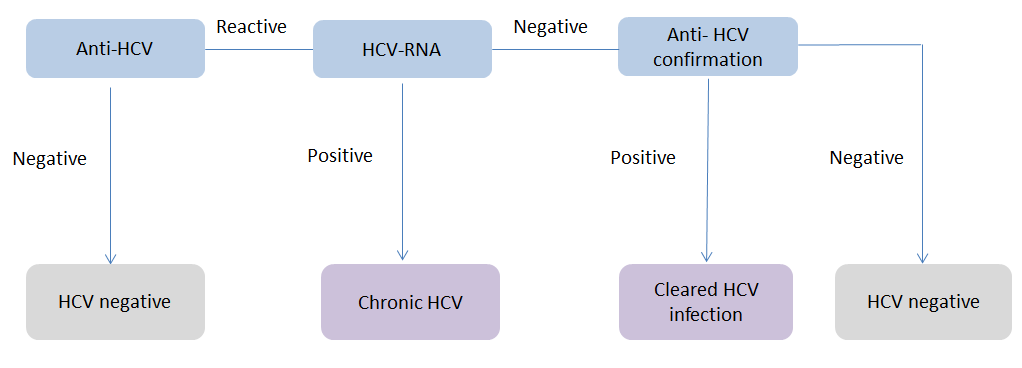


Number of participants per week during data collection period (5 September-16 November 2018)

1st invitation letter sent

2nd invitation letter sent

2nd press conference

(08.10.18)

1st press conference (05.09.18)

HCV prevalence

|  | | **n** | **Crude prevalence (%)** | **Weighted prevalence (%)** |
| --- | --- | --- | --- | --- |
| Anti-HCV (Elisa) (N=252) | Reactive | 2 | 0.8% [95% CI 0.2–3.1%] | 0.9% [95% CI 0.2–4.2%] |
|  | Negative | 250 |  |  |
| HCV RNA (N=2) | Positive | 2 |  |  |
|  | Negative | 0 |  |  |

Risk factors for HCV

| **Risk factors** | **n (%)** |
| --- | --- |
| Ever undergone surgery under general anesthesia | 161 (64.1%) |
| Ever undergone a blood transfusion (1992^a^) | 29 (11.7%) |
| Have (or have had) a body piercing | 22 (8.8%) |
| Have (or have had) a tattoo | 20 (8.0%) |
| Ever tried acupuncture | 12 (4.8%) |
| Close family diagnosed with hepatitis C | 10 (4.0%) |
| Ever used drugs? (Injected/snorted) | 4 (1/2) (1.6%) |
| Ever been imprisoned | 2 (0.8%) |
| Ever been through haemodialysis | 0 (0%) |
| Ever gone through an organ transplant | 0 (0%) |

*^a^ Routine testing of blood supply for hepatitis C began in 1992 in Bulgaria*

Age and sex among participants (n=252) versus total sample (n=1998)
